# Evaluation of a Probe-Based Enrichment Protocol for Nanopore Sequencing of Zoonotic Viruses

**DOI:** 10.3390/v17111465

**Published:** 2025-10-31

**Authors:** Kailin Hawes, Benjamin Greene, Zachary A. Weishampel, Paul A. Beare, Sarah van Tol, Paul Schaughency, Skyler Kuhn, Alison J. Peel, Vincent J. Munster, Claude Kwe Yinda

**Affiliations:** 1 Laboratory of Virology, Division of Intramural Research, National Institute of Allergy and Infectious Diseases, National Institutes of Health, Hamilton, MT 59840, USA; kbhawes902@gmail.com (K.H.); begreene16.2023@gmail.com (B.G.); zachary.weishampel@duke.edu (Z.A.W.); sarah.vantol@nih.gov (S.v.T.);; 2 Research Technologies Branch, Division of Intramural Research, National Institute of Allergy and Infectious Diseases, National Institutes of Health, Hamilton, MT 59840, USA; pbeare@niaid.nih.gov (P.A.B.);; 3 Genomics Research Section, Research Technologies Branch, Rocky Mountain Laboratories, National Institute of Allergy and Infectious Diseases, National Institutes of Health, Hamilton, MT 59840, USA; 4 Integrated Data Sciences Section, Research Technologies Branch, National Institute of Allergy and Infectious Diseases, National Institutes of Health, Bethesda, MD 20892, USA; 5 Sydney School of Veterinary Science, University of Sydney, Sydney, NSW 2050, Australia

**Keywords:** NGS, Oxford Nanopore sequencing, VirCapSeq-VERT, bioinformatics pipeline, enrichment

## Abstract

The detection of high-consequence viral pathogens is essential for spillover prevention and reduction in transmission but is limited by the low sensitivity of next-generation sequencing technology. Low-titer field samples from a variety of hosts are primarily composed of non-viral genomic material, reducing the probability of obtaining usable sequence data. Targeted enrichment, such as VirCapSeq-VERT, removes background genomic material to improve virus detection but is mainly used for sequencing clinical samples. We customized the VirCapSeq-VERT probe system to aid in the detection of zoonotic viruses of interest and adapted it for use on the Oxford Nanopore sequencing platform. We validated the method on a variety of samples, including a mock virome consisting of seven RNA viruses, samples from an animal laboratory study, and a set of animal field samples. We also developed Nanite, a lightweight bioinformatics pipeline, to perform bioinformatic analyses. Results indicated that the optimized enrichment protocol improved sequencing by enhancing the detection of viruses, increasing read lengths, and, in some cases, improving genomic coverage. Most importantly, the sequencing of zoonotic viruses was improved in field samples with low titers, suggesting that this protocol is a useful tool for increasing the efficacy of Oxford Nanopore sequencing for field-oriented applications.

## 1. Introduction

Nearly 70% of all human pathogens are zoonotic, and a disproportionately high number of those pathogens are RNA viruses. With zoonotic diseases on the rise, it is essential to establish robust protocols for detection of viruses in a variety of potential hosts to assist in spillover prevention efforts [1,2,3]. Polymerase chain reaction (PCR)-based methods have long been used for the detection of viruses, but these methods are limited by the relatively small number of viruses that can be probed per assay and may be affected by genetic drift or unrecognized diversity in viral populations [2,4,5,6,7]. For this reason, next-generation sequencing (NGS) technologies are an alternative that provide a variety of important benefits, including monitoring virus mutation and detecting novel viruses [2,8]. However, a limitation of NGS is that the high abundance of host genetic material and other background components relative to viral content results in a low proportion of viral reads during sequencing, posing challenges to effectively sequencing viruses [5,8]. Targeted enrichment protocols can resolve this issue by increasing the ratio of viral genomic material to background genomic material [9].

The virome capture sequencing platform for vertebrate viruses VirCapSeq-VERT is a targeted enrichment protocol that enriches vertebrate-infecting viral genomic material from samples [10]. In the protocol, Illumina libraries are prepared from samples before being hybridized with biotinylated probes designed against all viral taxa with at least one virus known to infect vertebrates. While highly effective, the Illumina-based VirCapSeq-VERT protocol requires complex laboratory workflows and specialized equipment that can limit its deployment in settings where rapid viral detection is most crucial. Oxford Nanopore sequencing offers a more accessible alternative in resource-limited areas compared to Illumina sequencing because of its portability and ease-of-use, making it a valuable tool for viral surveillance [4]. In addition, Oxford Nanopore technology allows for long-read sequencing, enabling singular reads to span nearly the entire viral genome [11].

Some targeted enrichment protocols have been adapted for Nanopore sequencing, including VirCapSeq-VERT, combining both comprehensive viral detection capabilities with accessibility and long read sequencing. A study by Pogka et al., [12] compared the performance of VirCapSeq-VERT on clinical samples prepared for Illumina and Nanopore sequencing, showing that both methods of sequencing offer enhanced viral detection with targeted enrichment. The major changes between the Illumina and Nanopore protocols are due to the increased heat sensitivity of Nanopore adapters compared to Illumina adapters [13]. Nanopore protocols thus require PCR cycles to be performed before adapter ligation, which becomes the final step to sequencing [13]. Most Nanopore-adapted VirCapSeq-VERT studies have demonstrated that enrichment improves viral sequencing in human clinical samples [10,14,15,16,17,18,19,20,21]. However, our aim is to develop an easily adjustable protocol to improve sequencing of RNA viruses of public health importance in non-clinical animal field samples. The Nanopore VirCapSeq-VERT protocol should be capable of detecting viruses in a variety of host animals, including those that may be asymptomatically infected, chronically infected, or expressing low viral titers [22].

Beyond sample preparation and sequencing, bioinformatic analysis of sequenced data is an essential step which may also be difficult in resource-limited settings. We developed a viral metagenomic analysis pipeline called Nanite to assist in identifying and classifying viral reads. Once installed, Nanite can be run on a local machine without relying on access to an HPC cluster, and it is simplified for users with a basic background in command line programming.

## 2. Materials and Methods

### 2.1. Development of VES_Viral_HyperExplore

An in-house customized probe set VES_Viral _HyperExplore was developed based on the publicly available VirCapSeq-VERT probe set [10] and produced by Roche (Indianapolis, IN, USA). Chosen viral families included *Arenaviridae*, *Coronaviridae*, *Filoviridae*, *Flaviviridae*, *Hantaviridae*, *Nidovirales*, *Orthomyxoviridae*, *Paramyxoviridae*, *Polyomaviridae*, *Poxviridae*, and *Togaviridae*. These families were chosen because they contain zoonotic viruses with high pandemic potential. See Appendix A for details on virus species and strain selection including accession numbers.

### 2.2. Samples

Three sets of samples were used:

#### 2.2.1. Mock Virome Composition

A mock virome was created using RNA from the following viruses: Ebola virus (EBOV), Lassa virus (LASV), Middle East respiratory syndrome coronavirus (MERS-CoV), severe acute respiratory syndrome coronavirus 2 (SARS-CoV-2), Menangle virus (MenPV), Hendra virus (HeV), and Nipah virus (NiV) (See Appendix B Table A1 for details). RNA was isolated from viral stock using the QIAamp Viral RNA Mini Kit (Qiagen, Germantown, MD, USA) according to manufacturer’s instructions. RNA copy numbers for each virus were determined via quantitative reverse transcription PCR (qRT-PCR) on a QuantStudio 3 Real-Time PCR System (Applied Biosystems, Waltham, MA, USA) using TaqMan Fast Virus 1-Step Master Mix for qPCR (Applied Biosystems, Waltham, MA, USA). See Appendix B Table A2 for a list of primers and probes and Appendix B Table A3 for thermocycling conditions and reagents. Once cycle threshold (Ct) -value was determined (Appendix B Table A4), RNA from each virus was pooled based on Ct, with the aim of creating a roughly equimolar mock virome pool. The amount of virus in the mock virome was then quantified with qRT-PCR using the same procedures as previously listed. Prior to targeted enrichment, mock virome samples were spiked with *Escherichia coli* (*E.coli*) RNA extracted via the RNeasy Mini Kit (Qiagen Germantown, MD, USA) according to the manufacturer’s protocol. Three samples were run at different ratios of viral RNA to *E.coli* RNA: 41 ng of viral RNA with no *E.coli* RNA (“high”), 33 ng of viral RNA with an estimated 40 ng of *E.coli* (“medium”), and 29 ng of viral RNA with an estimated 600 ng of *E.coli* (“low”). This aimed to examine the effectiveness of the target enrichment under various conditions, ranging from ‘least contaminated’ (high; with pure viral RNA) to ‘most contaminated’ field samples (low; with a low ratio of viral RNA to background RNA).

#### 2.2.2. Bat Experimental Samples

We utilized seven samples from a bat experimental infection study with *Artibeus jamaicensis* and filoviruses [23]. Briefly, RNA was extracted from oral swabs obtained from EBOV-infected *Artibeus jamaicensis* bats using the QIAmp Viral RNA Kit (Qiagen, Germantown, MD, USA) on the QIAcube HT automated extraction platform (Qiagen, Germantown, MD, USA) according to manufacturer’s instructions. In addition, qRT-PCR was performed as described [23]. Ct values of oral swabs collected on day 6 post-inoculation ranged from 27.2 to 35.9.

#### 2.2.3. Field Samples

Urine samples were collected from underneath flying fox (*Pteropus* spp. bat) roosts in Australia [24]. RNA was then isolated using the QIAmp Viral RNA Kit (Qiagen) on the QIAcube HT automated extraction platform (Qiagen, Germantown, MD, USA) according to manufacturer’s instructions. Samples were tested for the presence of MenPV using qRT-PCR on the QuantStudio 3 Real-Time PCR System (Applied Biosystems, Waltham, MA, USA, see Appendix B Table A3 for conditions). Six samples of RNA with Ct-values ranging from 30.6 to 37.3 were used in sequencing.

### 2.3. Targeted Enrichment and Sequencing

**Figure 1:**
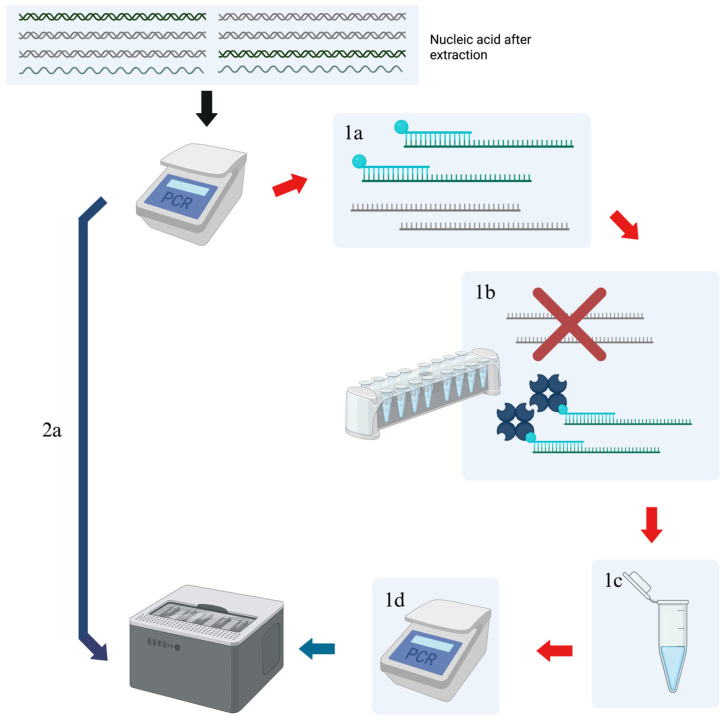
Comparison of untargeted and targeted sequencing strategies. Both protocols begin by amplifying genomic material by conducting PCR steps. In targeted enrichment, hybridization of probes to genomic materials follows the cleanup of PCR products (1a). The probes then bind to streptavidin beads (1b), which allows for washing of the sample on a magnetic rack to remove unwanted genomic material (1c). Another cycle of PCR steps is performed after purifying the sample for genomic material of interest (1d), and the resulting sample is cleaned up and prepared for sequencing. For untargeted samples, the initial PCR and subsequent cleanup are the only steps that precede sequencing (2a). Arrows indicate flow of steps of protocol. Red X indicate washed nucleic acid.

Untargeted libraries were prepared according to “Rapid viral metagenomics using SMART-9N amplification and nanopore sequencing” starting with the SMART-9n amplification step [25]. Briefly, random primers were annealed to template RNA during a short incubation period followed by snap cooling. Then, superscript IV reagents (Invitrogen, Carlsbad, CA, USA), RNase, nucleotides, and a template-switching oligonucleotide produced cDNA during a thermocycling stage. Taq polymerase and Oxford Nanopore barcodes were added to the cDNA, which then underwent 26 PCR cycles. PCR cleanup was performed with an equivalent ratio of AMPureXP beads (Beckman, Brea, CA, USA) to sample, and quantification was done with the Qubit High Sensitivity dsDNA assay kit (Invitrogen, Carlsbad, CA, USA). Once quantified, the samples were pooled in equimolar concentrations, and an Oxford Nanopore adapter was added for sequencing. The Oxford Nanopore flow cell was primed with flush buffer and flush tether before adding the sample mixture which contained DNA, sequencing buffer, and loading beads. For targeted enrichment, the same protocol was used but included an additional capture protocol following only 24 cycles of PCR and subsequent cleanup. For the enrichment capture method, KAPA HyperCap 3.4 kit and protocol (Roche, Indianapolis, IN, USA) were used. Briefly, the protocol began by preparing samples with COT Human DNA, KAPA Hybrid Enhancer, and KAPA HyperPure Beads. Once bead wash was performed, Universal Enhancing Oligos and Hybridization Master Mix were added. Hybridization Master Mix contained buffer, component H, and PCR grade water. Eluate was then transferred to a tube containing KAPA HyperCap Target Enrichment Probes. The sample was subjected to 20 h capture hybridization at 55 °C. After hybridization, the samples were cleaned by a series of wash buffers and capture beads. The cleaned, enriched PCR products were subjected to Nanopore library preparation using the following procedure: reaction mixture contained 25 µL of LongAmp Taq 2× Master Mix (New England Biolabs, Ipswich, MA, USA), 0.5 µL of RLB 01-12 (Oxford Nanopore Technologies, Oxford, UK), 20 uL of nuclease-free water, and 5 µL of cleaned, hybridized DNA. The cycling conditions in the Bio-Rad C1000 Touch 96-well PCR Thermal Cycler (Bio-Rad, Hercules, CA, USA) were as follows: 95 °C for 45 s, followed by 24 cycles of 95 °C for 15 s, 56 °C for 15 s, and 65 °C for 5 min, with a final hold of 65 °C for 10 min. Post-PCR cleanup was performed with KAPA HyperPure Beads followed by quantification of DNA using the Qubit High Sensitivity dsDNA assay kit (Invitrogen, Carlsbad, CA, USA). Libraries were prepared and loaded onto an Oxford Nanopore GridION using a FLO-MIN114 flow cell (Oxford Nanopore Technologies, Oxford, UK) as described before [26]. A flow diagram for the enrichment procedure is summarized in Figure 1.

### 2.4. Sequence Analysis

We developed Nanite [27], a free and open-source viral metagenomic analysis pipeline designed specifically for the analysis, assembly, and classification of Oxford Nanopore sequencing data (available at https://github.com/OpenOmics/nanite, accessed on 5 October 2024). Nanite is an easy-to-use and highly reproducible pipeline that is optimized to run on standard consumer-grade hardware, such as a laptop in the field or a local desktop computer. In its assembly-free mode, Nanite is capable of processing a large number of samples without the need for specialized hardware or a high-performance computing cluster. The pipeline incorporates key data-processing steps, including read filtering, optional genome assembly, alignment, and taxonomic classification.

The pipeline begins by filtering low-quality reads using NanoFilt [28]. The filtered reads are then aligned to NCBI’s viral database using minimap2 [29], facilitating the identification of viral genomes. For taxonomic classification, Nanite generates an interactive report of the aligned reads using Krona [30], which provides a visual representation of the taxonomic composition. Nanite optionally supports a genome assembly step to recover high-quality viral genomes. In this mode, filtered reads are assembled into contigs using Flye [31,32], and high-quality viral genomes are recovered using ViralFlye [33]. When this assembly option is provided, Nanite produces an additional interactive report using the assembly outputs. This can be used to visualize the taxonomic classification of assembled reads alongside unassembled reads.

Additionally, an in-house custom pipeline called VirView (available at https://github.com/greenehurls/VirView, accessed on 5 October 2024) was developed to streamline the analysis of sequences for individual viruses and included the analysis of genomic coverage, read length, and read quantity using Minimap2 [29] and SAMtools [34].

### 2.5. Statistical Analysis

Wilcoxon matched-pairs signed rank tests for nonparametric data were performed to compare differences between sample performance with and without targeted enrichment on GraphPad Prism version 10.4.1.

## 3. Results

**Figure 2:**
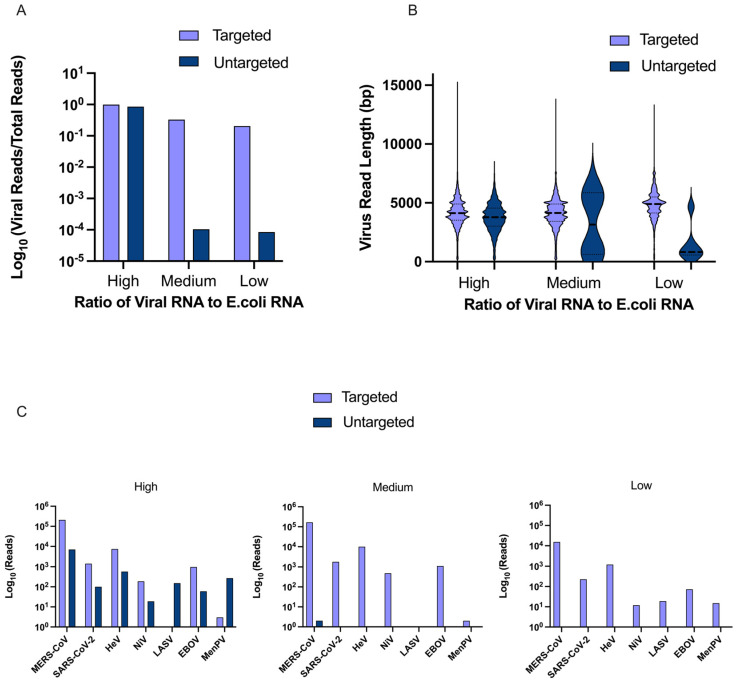
Use of a mock virome to explore efficacy of targeted enrichment protocol. (**A**) Results comparing the number of normalized viral reads between targeted and untargeted mock virome samples at high, medium, and low concentrations of viral RNA on a log scale. (**B**) A truncated violin plot comparing virus read length in bp between targeted and untargeted mock virome samples at high, medium, and low concentrations of viral RNA. (**C**) Individual graphs for mock virome samples with high, medium, and low concentrations of viral RNA comparing the log number of reads per virus with and without targeted enrichment. Note, the absence of a bar indicates no reads with the exception of the low concentration untargeted sample having one MERS-CoV read not visualized on the graph.

The mock virome consisted of seven RNA viruses of interest, including EBOV, LASV, MERS-CoV, SARS-CoV-2, MenPV, HeV, and NiV. Ct-values of the mock virome ranged from 25.5 to 31.0 (Appendix B Table A4). Three different mock virome samples were run to validate the efficacy of the sequencing protocol at decreasing ratios of viral RNA to a background contaminant, in this case *E.coli* RNA (high, medium and low ratios of viral RNA to *E.coli* RNA). For samples with high relative concentrations of viral RNA, the normalized viral reads (number of viral reads divided by the total number of reads from the sequencing run) were comparable for samples with and without targeted enrichment. However, in samples with low and medium relative concentrations of viral RNA, targeted enrichment produced a fold increase in normalized viral reads of over 2000× and 3000×, respectively (Figure 2A). In both untargeted samples with *E.coli*, over 99% of all reads were bacterial in origin, while in targeted samples with *E.coli*, bacterial reads reduced to around 67% to 79% of total reads. The average viral read length of untargeted mock virome samples ranged from 1468 to 3754 bp per read, while the average viral read length for targeted mock virome samples ranged from 4096 to 4794 bp per read. Independent of viral RNA concentration, targeted enrichment also increased the average viral read length and allowed for a set of long reads relative to viral genome length, the maximum being 15,097 bp mapping to MERS-CoV (Figure 2B). At a high concentration of viral RNA, all viruses were detected in both targeted and untargeted samples, and the targeted enrichment increased the number of reads per virus for all viruses except LASV and MenPV. At medium or low concentrations of viral RNA, where most reads represented amplification of bacterial RNA, the beneficial effect of targeted enrichment was more pronounced. With a medium concentration of viral RNA, untargeted samples only contained two reads for MERS-CoV, lacking data for all other viruses, whereas targeted enriched samples provided data for six of the seven viruses present in the mock virome, only missing LASV. Similarly, in untargeted mock virome samples with low concentrations of viral RNA, only one read matched to MERS-CoV, whereas all seven viruses were identified in the corresponding targeted sample (Figure 2C). Despite the mock virome PCR data indicating relatively even Ct-values of viruses, MERS-CoV made up the majority of viral reads, about 72% on average, while NiV and MenPV only accounted for, on average, 0.1% and 0.3% of viral reads, respectively.

**Figure 3:**
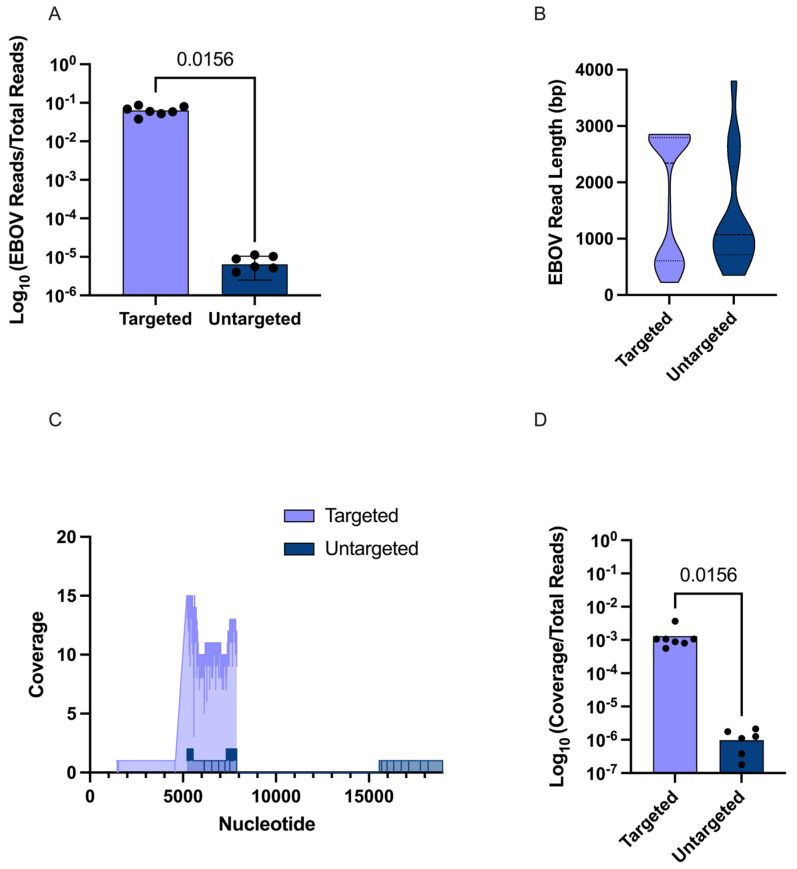
Sequencing and targeted enrichment of EBOV from swab samples. (**A**) Results comparing the number of normalized EBOV reads between targeted and untargeted samples on a log scale. (**B**) A truncated violin plot comparing EBOV read lengths in bp between targeted and untargeted samples. (**C**) A coverage graph of the EBOV genome for a single sample to compare depth and breadth of coverage in targeted versus untargeted samples. (**D**) Results comparing the normalized coverage between targeted and untargeted samples on a log scale.

Next, we sought to assess the performance of the method in animal-based samples, using swab samples from animal studies conducted in a laboratory setting. We used EBOV positive swab samples from *Artibeus jamaicensis* bats with high Ct values (range: 27.2–35.9) and lack of overt clinical disease, representing samples that would potentially benefit from the use of this method. While only minimal sequencing data could be collected for the untargeted EBOV swab samples, the targeted EBOV swab samples showed a significant increase of nearly 10,000-fold in normalized reads (Figure 3A). Though very different in normalized quantity, the untargeted and targeted samples had similar average viral read lengths of 1394 and 1729 bp per read, respectively (Figure 3B). The EBOV genomic coverage was higher in three of the seven untargeted samples compared to targeted counterparts, but the depth of coverage was significantly higher in targeted samples (Figure 3C). On average, untargeted samples had 13% coverage while targeted samples had 14% coverage, with the highest coverage being 32% in an untargeted sample. It is possible that this deviation from expected results in terms of increased coverage in three untargeted samples could be attributed to poor sequencing quality, as the sequencing run yielded a lower than ideal number of reads. However, as these samples were derived from a separate study with limited remaining material, re-sequencing was not feasible to confirm this hypothesis. Nevertheless, average normalized genomic coverage of EBOV was significantly higher with targeted enrichment, showing a nearly 3000-fold increase (Figure 3D). Based on coverage per bp, the average depth was 0.83 reads/bp without targeted enrichment and 7.75 reads/bp with targeted enrichment.

**Figure 4:**
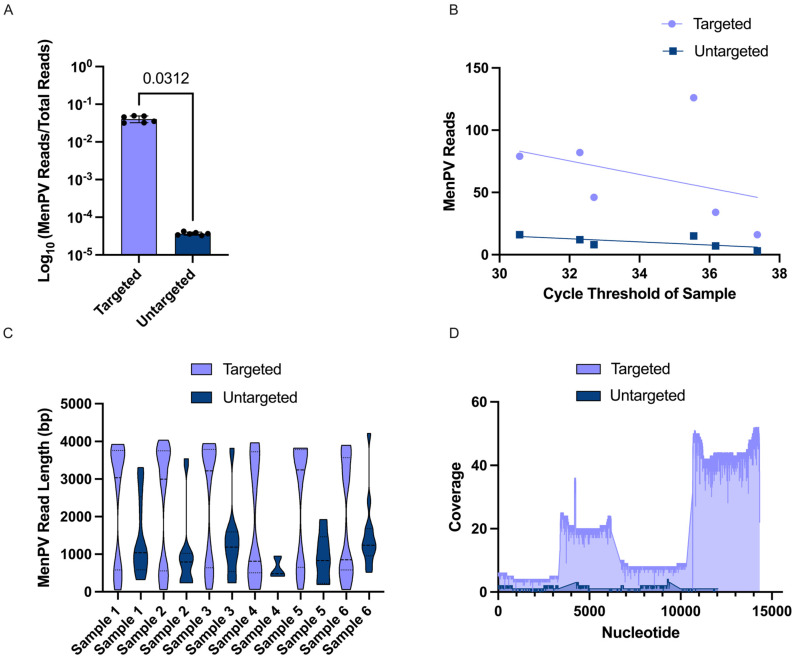
Sequencing and targeted enrichment of field samples positive for MenPV from Australian Pteropus bats. (**A**) A comparison of the normalized MenPV reads for six different samples with and without targeted enrichment on a log scale. (**B**) A plot of the number of MenPV reads per sample versus the Ct value of that sample with a separate simple linear regression for targeted and untargeted samples. (**C**) A truncated violin plot comparing the MenPV read length in bp of six different samples treated with and without targeted enrichment. (**D**) A coverage graph of the MenPV genome for a single sample to compare depth and breadth of coverage in targeted versus untargeted samples.

Once verified on laboratory-based animal samples, the final step was to ensure the platform could enhance the detection of viruses in animal-based field samples. The field samples that were sequenced had previously tested positive for MenPV in qRT-PCR, with Ct values ranging from 30.6 to 36.4. Each sample was run with and without targeted enrichment to directly compare the effect on sequencing. Targeted enrichment resulted in an increase in the average number of MenPV reads per sample from 10.17 to 63.83 and a significant increase in average normalized MenPV reads of around 1100× (Figure 4A). In both targeted and untargeted samples there was a negative correlation between Ct value and the number of viral reads (Figure 4B). In terms of viral reads, the enriched samples had significantly higher average lengths than the untargeted samples (Figure 4C, Table A5). In addition to an increase in viral reads and read length, both the breadth and depth of MenPV coverage were statistically higher in targeted samples. For sample 2, which had the highest number of viral reads in targeted sequencing, the average depth across the MenPV genome was only 0.82 reads/bp for the untargeted run compared to 16.76 reads/bp for the targeted sample. Although neither version of sample 2 had complete coverage across the genome, the untargeted sequencing results had 56% genomic coverage while the targeted results had 86% genomic coverage (Figure 4D).

## 4. Discussion

Oxford Nanopore technology is a relatively low-cost sequencing platform, especially in regions of the world that lack resources for higher input sequencing technology [4]. However, animal samples are often heavily contaminated by host and bacterial DNA, resulting in a small minority of genomic data mapping to viruses, of which most are bacteriophages and not viruses of interest [35]. Targeted enrichment has emerged as a promising technique for resolving these issues of contamination [5], however challenges still remain with applying this approach to long-read sequencing and in resource-poor environments. To address this, we sought to adapt a virus-specialized VirCapSeq-VERT [10] targeted enrichment technique to the Oxford Nanopore technology to improve capabilities to monitor and detect high-consequence pathogens from reservoir hosts. The newly customized VES_Viral_HyperExplore probe set was designed for a variety of emerging zoonotic RNA viruses of interest. The list of viruses included in the probe set can evolve as pathogens of interest diverge genetically or as novel pathogens emerge in animal and human populations. The tested probe set and related protocol proved effective in increasing the yield of viral sequences of interest, both from a mock virome and from swab and urine samples. In addition, we successfully developed a new bioinformatics pipeline called Nanite to facilitate Oxford Nanopore data analysis in low resource settings. Nanite successfully identifies viral reads in sequence data, indicating which viral populations are present as well as compiling all viral reads for optional further analysis.

The success of targeted enrichment became more evident as the concentration of viral RNA decreased, indicating that this protocol is well suited for our aims to sequence field samples with low viral loads. At the lowest concentration of viral RNA, targeted enrichment allowed for successful amplification of all seven viruses and produced full genomes of three of the viruses in the mock virome, including MERS-CoV at a depth of around 6500×, SARS-CoV-2 at a depth of over 100×, and HeV at a depth of nearly 850×. This highlights its potential compared to unbiased sequencing, which detected only MERS-CoV at low concentrations of viral RNA. However, we observed that although the mock virome PCR data suggested relatively even Ct values across viruses, the sequencing output showed considerable variation in reads per virus. This variation likely arises from three factors: first, inherent inequality in sequencing, where even identical RNA or DNA concentrations rarely yield the same number of reads; and second, differences in viral Ct values across assays, which are not fully comparable and can result in some viruses being present at higher concentrations than others. In addition, the designed probes may not be equally effective in binding RNA, potentially leading to bias in which viral genomes are replicated.

The protocol’s effectiveness was also confirmed using swabs from EBOV-infected *Artibeus* bats, where low-level viral shedding in the absence of overt clinical symptoms mirrored conditions expected when studying natural reservoir species. Spillover pathways between reservoir hosts and humans require the reservoir host to be infected with the zoonotic pathogen, and the pathogen must be released from the host, usually through excretion in urine, feces, or saliva [36,37,38]. Reservoir species may not exhibit signs of disease, as is suspected in the case of Marburg virus, Hendra virus, and Nipah virus in their natural hosts. In addition, viral shedding may be intermittent with both prevalence and viral load amplified by periods of stress, potentially leading to spillover events [36,39,40]. Improvements in normalized reads and depth of coverage of EBOV RNA extracts with targeted enrichment highlights the platform’s applicability to samples with low viral loads. However, the higher genomic coverage in some untargeted samples over targeted samples indicates a potential variability in the protocol with very low viral load samples. These results suggest that although VirCapSeq-VERT performs well as a detection tool, additional protocol optimization is needed to reliably achieve full genome recovery in samples with low viral loads.

Sequencing of urine collected from roosts of Australian *Pteropus* bats provided a final validation of the viability of the method for use with field samples. Urine collection from plastic sheeting placed underneath bat roosts has been an effective method to screen for bat-borne viruses at the population level [24,41]. Higher normalized read counts, longer reads, and greater depth and breadth of coverage of MenPV from urine samples with targeted enrichment demonstrates the method’s applicability to field samples where viral titers are low. While we have demonstrated that the probe-based enrichment protocol enhances viral detection in the sample types used, further evaluation is needed to determine the capacity of the protocol in a wider variety of samples, including fecal or environmental samples in addition to samples with a greater complexity of non-viral genomic material.

Most available hybrid capture methods are designed for use on Illumina Sequencing platforms [42]. Additionally, most protocols are targeted at clinical samples rather than field specimens [14,16,17,18,19,21]. By establishing hybrid capture using Oxford Nanopore technology on field samples, this study effectively bridged that gap. Although Oxford Nanopore Technology reduces infrastructural requirements and offers cost-effectiveness [43], bioinformatic analysis continues to pose a major challenge for genomic surveillance efforts. The predominance of Linux-based tools [44] creates barriers in settings where Windows operating systems are more widely available, particularly in low-resource environments. Consequently, sequencing data often must be transferred to centralized facilities with advanced IT infrastructure [45], introducing logistical challenges and delays in data processing. By contrast, Nanite provides a lightweight, Windows-optimized solution that minimizes computational demands and reduces the need for specialized expertise. This enables analysis to be performed closer to the point of sample collection, thereby shortening turnaround times. The deployment of such tools has important implications for expanding the reach of genomic surveillance and democratizing access to pathogen sequencing capacity in under-resourced settings.

## 5. Conclusions

The study demonstrates that targeted enrichment significantly improves the sensitivity of Oxford Nanopore sequencing for detecting high-consequence viral pathogens in field samples. By customizing the VirCapSeq-VERT probe system tailored with a novel bioinformatics pipeline Nanite, we showed enhanced viral read recovery, even for low-titer zoonotic viruses. These findings suggest that this optimized approach could be a valuable tool for field-based viral surveillance, addressing the limitations of next-generation sequencing in low-titer samples.

## Data Availability

The original contributions presented in this study are included in the article/Supplementary Material. Further inquiries can be directed to the corresponding author.

## Supplementary Materials

The following supporting information can be downloaded at: https://www.mdpi.com/article/10.3390/v17111465/s1, Table S1: Virus sequences included in the VES_Viral_HyperExplore probe set (Appendix A).

## Appendix A

Virus sequences included in the VES_Viral_HyperExplore probe set: See attached excel file.

## Appendix B

**Table A1:** Strain information for viruses in mock virome.

Virus	Strain
EBOV	Zaire ebolavirus Kikwit (15476)
HeV	Hendra henipavirus/Pteropus spp/Australia/RML-084/2018
NiV	Nipah henipavirus/Pteropus-medius/Bangladesh/RML-EHA-08/2018
MERS-CoV	Domedary camel MERS-CoV BF785
SARS-CoV-2	2019-nCoV/USA-WA1/2020 (GenBank Accession MN985325)
LASV	LASV Macenta wt
MenPV	Menangle virus from bat field samples

**Table A2:** Primers and probes for qRT-PCR.

Virus	Forward Primer	Reverse Primer	Probe
HeV/NiV	CAGATGACATACAACTGGACCCA (50 µM)	CCCTCTCTCAGGGCTTGAAG (50 µM)	SUN-GTATACCATGATCATGGAGGAGAATGTACC-TAMRA (13.9 µM)
LASV	CCACCATYTTRTGCATRTGCCA	GCACATGTNTCHTAYAGYATGGAYCA	FAM-AARTGGGGYCCDATGATGTGYCCWTT-ZEN-IBHQ
MERS-CoV	CTATCTCACTTCCCCTCGTTCTC (2 µM)	GGAAGATGGCCATAGGAGCC (2 µM)	FAM-CTGAGGCGCAGATTATTGCC-ZEN-IBHQ (2 µM)
EBOV	CAGCCAGCAATTTCTTCCAT (20 µM)	TTTTCGGTTGCTGTTTCTGTG (20 µM)	FAM-TCATTGGCGTACTGGAGGAGCAGG-ZEN-IBHQ (5 µM)
SARS-CoV-2	AACAGGTACGTTAATAGTTAATAGCGT (20 µM)	ATATTGCAGCAGTACGCACACA (20 µM)	FAM-ACACTAGCCATCCTTACTGCGCTTCG (5 µM)
Menangle-like viruses	ATCTGGATTCCCCTATAGTG (20 µM)	CTAGGTTAGGGAAGTATGAGTTC (20 µM)	FAM-CCAAAAGTTAGGCCAGTTACCTGG-ZEN-IBHQ (5 µM)

**Table A3:** Conditions for qRT-PCR.

Virus	Reagents	Thermocycling Conditions
EBOV, SARS-CoV-2	1 µL of primers and probes 5 µL of master mix 9 µL of nuclease-free water 5 µL of RNA	10 min 50 °C 5 min 95 °C 40×: 10 s 95 °C 30 s 60 °C
HeV, NiV, MERS-CoV	0.5 µL of primers and probes 5 µL of master mix 4.5 µL of nuclease-free water 5 µL of RNA	5 min 50 °C 20 s 95 °C 40×: 15 s 95 °C 60 s 60 °C
LASV	0.5 µL of primers and probes 5 µL of master mix 9.5 µL of nuclease-free water 5 µL of RNA	5 min 50 °C 20 s 95 °C 40×: 15 s 95 °C 60 s 60 °C
Menangle virus	0.5 µL of primers and probes 5 µL of master mix 9.5 µL of nuclease-free water 5 µL of RNA	10 min 50 °C 5 min 95 °C 40×: 10 s 95 °C 30 s 60 °C

**Table A4:** Ct values for viruses in mock virome.

Virus	Ct Value in Mock Virome
HeV	28.286
NiV	28.279
LASV	26.927
MERS-CoV	27.795
EBOV	31.022
SARS-CoV-2	26.75
MenPV	25.519

**Table A5:** Average viral read lengths in MenPV samples.

Sample Number	Targeted Average Viral Read Length	Untargeted Average Viral Read Length
Sample 1	2361	1442
Sample 2	2277	1003
Sample 3	2375	1366
Sample 4	1816	613
Sample 5	2441	929
Sample 6	1846	1514
